# Neural Correlates of Repetition Priming: A Coordinate-Based Meta-Analysis of fMRI Studies

**DOI:** 10.3389/fnhum.2020.565114

**Published:** 2020-09-18

**Authors:** Sung-Mu Lee, Richard N. Henson, Chun-Yu Lin

**Affiliations:** ^1^Taiwan International Graduate Program in Interdisciplinary Neuroscience, National Cheng Kung University and Academia Sinica, Taipei, Taiwan; ^2^MRC Cognition and Brain Sciences Unit, University of Cambridge, Cambridge, United Kingdom; ^3^Department of Psychiatry, University of Cambridge, Cambridge, United Kingdom; ^4^Department of Psychology, National Cheng Kung University, Tainan, Taiwan

**Keywords:** repetition priming, meta-analysis, fMRI, perceptual, conceptual, stimulus-response bindings

## Abstract

Repetition priming is a form of implicit memory, whereby classification or identification of a stimulus is improved by prior presentation of the same stimulus. Repetition priming is accompanied with a deceased fMRI signal for primed vs. unprimed stimuli in various brain regions, often called “repetition suppression,” or RS. Previous studies proposed that RS in posterior regions is associated with priming of perceptual processes, whereas RS in more anterior (prefrontal) regions is associated with priming of conceptual processes. To clarify which regions exhibit reliable RS associated with perceptual and conceptual priming, we conducted a quantitative meta-analysis using coordinate-based activation likelihood estimation. This analysis included 65 fMRI studies that (i) employed visual repetition priming during either perceptual or conceptual tasks, (ii) demonstrated behavioral priming, and (iii) reported the results from whole-brain analyses. Our results showed that repetition priming was mainly associated with RS in left inferior frontal gyrus and fusiform gyrus. Importantly, RS in these regions was found for both perceptual and conceptual tasks, and no regions show RS that was selective to one of these tasks. These results question the simple distinction between conceptual and perceptual priming, and suggest consideration of other factors such as stimulus-response bindings.

## Introduction

Priming refers to behavioral changes in the identification, production, or classification of a stimulus caused by a previous encounter with the same or a similar stimulus (Tulving and Schacter, 1990). Priming has been associated with implicit, or unconscious, memory, because it is normally unaffected by brain lesions that cause impairments of conscious or explicit memory, i.e., amnesia (Gabrieli et al., 1995; Keane et al., 1995), and because it is measured by tasks that do not require explicit memory for the previous encounter. Over the last few decades, the neural bases of priming have been studied extensively with functional magnetic resonance imaging (fMRI). Specifically, fMRI studies found that repeated processing of a primed stimulus is associated with decreased responses in various brain regions. This phenomenon is called repetition suppression (RS; for review, see Grill-Spector et al., 2006). RS has therefore been proposed as a neural signature of behavioral priming (e.g., Schacter and Buckner, 1998; Wiggs and Martin, 1998; Henson, 2003; Gotts et al., 2012), though in fact direct correlations between the size of RS and the amount of behavioral priming are rarely reported (Schacter et al., 2007), and when they are found, such behavioral correlations are mainly with RS in frontal brain regions (e.g., Dobbins et al., 2004; Maccotta and Buckner, 2004; Horner and Henson, 2008).

Kim (2016) performed a meta-analysis of fMRI studies to clarify which brain regions exhibit reliable RS. A meta-analytic approach is important to account for different statistical thresholds across studies, and potentially to generalize over differences in stimuli and task. Kim collected 1,020 coordinates of peak foci from 106 experiments and 1,454 participants. The results showed consistent RS in bilateral inferior frontal cortex and ventral occipitotemporal cortex. Kim (2016) suggested that RS in ventral occipitotemporal cortex reflects facilitated perceptual processing (since all the experiments used visual stimuli), while RS in inferior frontal cortex reflects some combination of facilitated conceptual processing, automatized stimulus-response mapping, less demand for top-down enhancement, and reductions in novelty. Additionally, Kim found that most regions showing RS for one type of visual stimulus also showed RS for other types. For example, direct comparisons between words, non-words, scenes, faces, and objects indicated that ventral occipitotemporal cortex shows RS regardless of type of visual stimulus.

In addition to RS, repeated stimulus presentation can result in enhanced brain responses relative to its first presentation, or “repetition enhancement” (RE). While RE is most often reported in brain regions associated with explicit memory, most likely reflecting incidental conscious memory for the previous encounter with a stimulus (even though that encounter is irrelevant to performance of priming tasks), it has occasionally been reported in brain regions associated with priming, and which show RS under other conditions (see Schacter et al., 1995; Dolan et al., 1997; George et al., 1999; Henson et al., 2000).

However, Kim's (2016) meta-analysis searched for the terms “repetition suppression” and “neural priming” to identify initial candidate articles, many of which may not have reported concurrent behavioral priming. As far as we are aware, the only meta-analyses of behavioral priming and its neural correlates have focused on specific priming paradigms, such as negative priming (e.g., a slowing rather than speeding of responses to repeated stimuli; Yaple and Arsalidou, 2017), semantic priming (e.g., speeded responding to a target preceded by a semantically related prime; Rodd et al., 2015) and processing of subliminal stimuli (Brooks et al., 2012; Meneguzzo et al., 2014). Therefore, the first goal of the present study was to restrict the meta-analysis to studies that reported significant behavioral priming (in implicit memory tasks), but expand this across a range of different repetition priming paradigms (such as speeded classification, item identification, and word-stem completion, but excluding negative priming, semantic priming, and masked/subliminal priming), and to allow for RE as well as RS. Like Kim, we used activation likelihood estimation (ALE; Eickhoff et al., 2009, 2012; Turkeltaub et al., 2012) to determine the convergence of statistical peaks showing RS and RE.

Furthermore, we distinguished two different types of repetition priming (Schacter and Buckner, 1998): perceptual priming and conceptual priming. Perceptual processing relies on the physical characteristics of the stimuli, or “data-driven” processes, whereas conceptual priming relies on the meaning of the stimuli, or “conceptually-driven” processes (Jacoby, 1983; Blaxton, 1989). Since we examined only repetition priming but not semantic, relatedness or associative priming in this meta-analysis, perceptual priming denotes “repetition priming in perceptual tasks” and conceptual priming denotes “repetition priming in conceptual tasks.” Examples of commonly-used perceptual priming tasks include perceptual identification (naming words or objects as quickly as possible) and word-stem/fragment completion (producing the first word that comes to mind in response to a cue representing part of that word); examples of conceptual priming tasks include category generation/association (generating several exemplars of a cued category) and conceptual classification (e.g., deciding as quickly as possible whether an object is living or non-living). Numerous psychological studies have reported behavioral dissociations between these two types of priming (e.g., in terms of their sensitivity to other psychological factors; see Roediger and McDermott, 1993 for review).

Early neuropsychological support for a neural dissociation between perceptual vs. conceptual priming arose in patients with Alzheimer's disease (AD). While AD can damage many areas of the brain, particularly as the disease progresses, the damage is normally greatest in medial temporal lobe structures, and is least in early sensory regions. Consistent with this, AD patients generally show intact perceptual priming (Buckner et al., 1995; Fleischman et al., 1995; Gabrieli et al., 1995; Keane et al., 1995) despite defective conceptual priming (Salmon et al., 1988; Monti et al., 1996; Gabrieli et al., 1999). However, the preservation of conceptual priming in AD has been reported in some studies (Grosse et al., 1990; Meiran and Jelicic, 1995; Fleischman and Gabrieli, 1998). These discrepancies might reflect differences in task characteristics, or owe to the highly variable and diffuse extent of pathology in AD, which also depends on the stage of the progressive neurodegenerative disease (Fleischman et al., 2005; Fleischman, 2007). Other studies have examined patients with more focal, acquired lesions. For example, Gong et al. (2016) used a picture identification task (i.e., perceptual priming) and a category exemplar generation task (i.e., conceptual priming) to study patients with occipital vs. frontal lobe lesions. They found that the performance of perceptual priming was poorer in occipital lesion group, whereas the performance of conceptual priming was poorer in frontal lesion group. Thus, these neuropsychological studies support a double dissociation, with occipital cortex supporting perceptual priming and frontal cortex supporting conceptual priming.

Neuroimaging studies have provided some further support for the claim that perceptual and conceptual priming are associated with different brain regions (Schacter and Buckner, 1998; Schacter et al., 2007). As might be expected from the component process view (Schacter and Buckner, 1998; Henson, 2003), according to which RS reflects facilitation of specific processes involved in a given task, perceptual priming tends to produce RS in more sensory-related regions, such as occipitotemporal gyrus for visual stimuli, while conceptual priming tends to produce RS in frontotemporal regions (e.g., inferior frontal gyrus and inferior temporal cortex). Moreover, some studies have found correlations between the size of the behavioral priming effect in conceptual priming tasks and the amount of RS in inferior frontal gyrus (Lustig and Buckner, 2004; Maccotta and Buckner, 2004; Bunzeck et al., 2006; Turk-Browne et al., 2006; Soldan et al., 2010) or fusiform gyrus (Turk-Browne et al., 2006; Soldan et al., 2010), though such correlations are not always reported. Interestingly, these correlations are normally found with RS in inferior frontal gyrus and conceptual priming, as measured by reduced RTs in semantic classification tasks; we are not aware of any study that has found a correlation between RS in occipitotemporal areas and measures of perceptual priming, e.g., reduced RTs in perceptual priming tasks.

An alternative to the component process view of RS is the hypothesis that RS reflects retrieval of stimulus-response (S-R) bindings (for review, see Henson et al., 2014). According to this view, the response made on the initial stimulus is bound to that stimulus, such that when the stimulus is repeated, the response can be generated without repeating the perceptual and conceptual component processes that were initially engaged to produce that response, resulting in reduced activity in relevant regions. On the contrary, if S-R bindings are no longer relevant, those brain regions are equal active for repeated and initial processing, causing an absence of RS. The first neuroimaging evidence for this possibility came from Dobbins et al. (2004), who showed that simply reversing the task in a semantic classification paradigm abolished RS in fusiform and frontal cortex. Their argument was that responding to the first presentation of a stimulus creates a unique S-R binding, such that when that stimulus is primed, the S-R binding can be retrieved and used to generate the response, bypassing the need for repeated perceptual or conceptual processing. Subsequent studies (e.g., Horner and Henson, 2008, 2011; Race et al., 2008) showed that task reversal (or other ways to reduce the influence of S-R bindings) does not always abolish RS in occipitotemporal regions, though it invariably does so in frontal regions (and indeed, sometimes reversing the S-R contingency can produce RE rather than RS in frontal regions; Horner and Henson, 2011). While the contributions of S-R bindings do not rule out a role for occipiotemporal cortex in perceptual priming or inferior frontal cortex in conceptual priming, they do confound many behavioral measures of priming (particularly those using speeded classification tasks; Horner and Henson, 2009), and it is possible that the correlations found between RS in inferior frontal gyrus and priming in conceptual priming tasks actually reflects retrieval of S-R bindings, i.e., does not reflect facilitated conceptual processing. Thus, the second goal of our meta-analysis was to see whether the brain regions showing RS differed according to perceptual vs. conceptual priming, or if not, to consider whether a common pattern of results could owe to S-R bindings.

Our final goal relates to the nature of the stimuli used. Many studies assume they are measuring conceptual priming because they use a task that requires conceptual processing, even though the stimuli are also repeated in the same perceptual format, and therefore the neural and/or behavioral consequences of priming can include additional perceptual processes. To isolate “purer” conceptual priming, some studies have examined priming and RS across a change in the perceptual format (e.g., from pictures to words, e.g., Simons et al., 2003). Therefore, we performed a sub-analysis of conceptual priming studies in which different exemplars of stimuli were used for primed vs. unprimed conditions (even though the same “concept” was repeated).

In sum, the three objectives of this meta-analysis are (1) to identify the brain regions with the most consistent RS and RE across fMRI studies that show concurrent behavioral priming, (2) to test whether different brain regions show RS for perceptual vs. conceptual priming, and (3) to further examine the RS associated with conceptual priming across stimuli with minimal perceptual overlap.

## Materials and Methods

This study followed Preferred Reporting Items for Systematic Reviews and Meta-Analyses (PRISMA) guidelines (Moher et al., 2009).

### Literature Search and Selection Criteria

**Figure 1:**
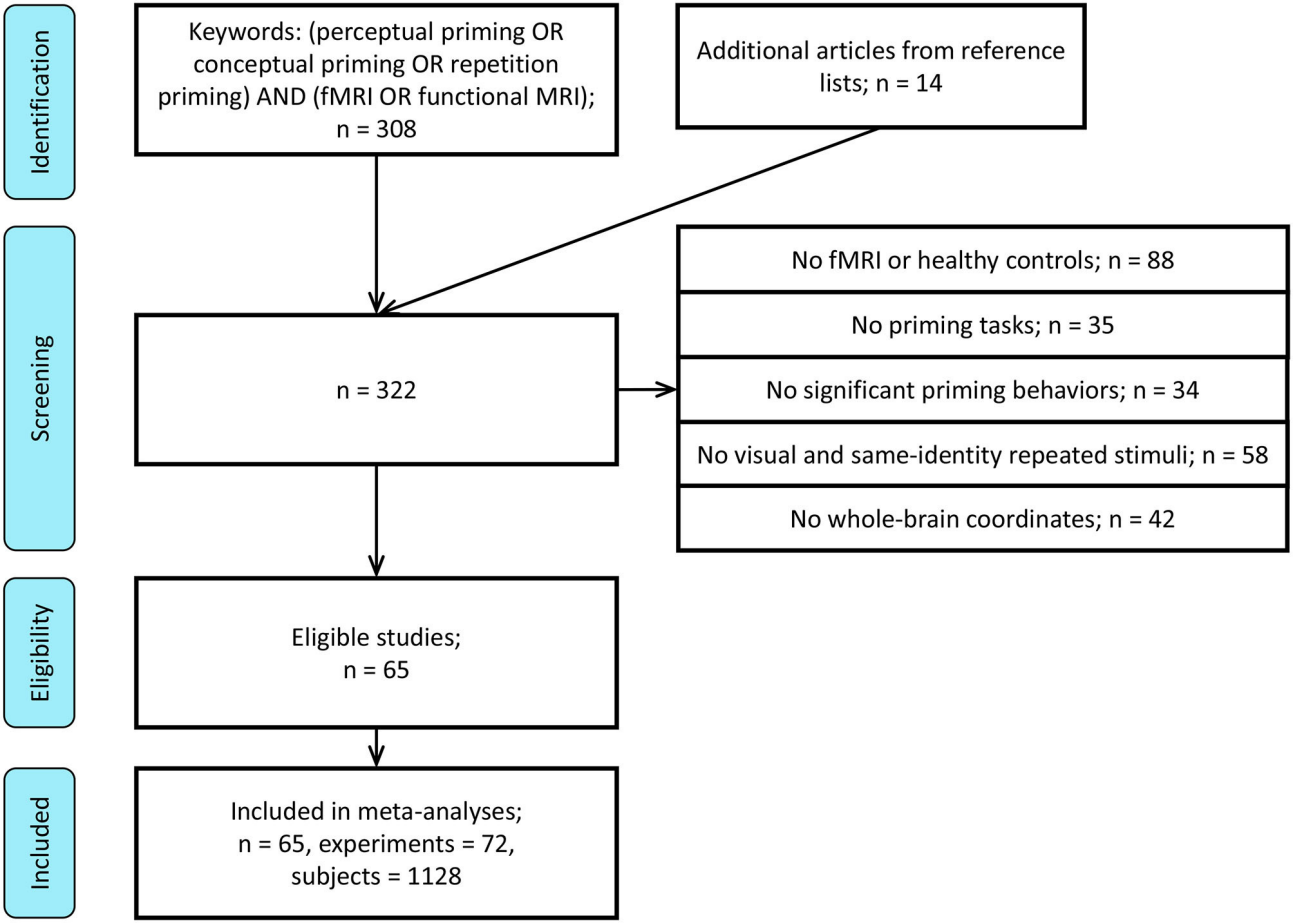
PRISMA flow chart for procedure of study selection.

A literature search for human fMRI studies on behavioral priming up to October 31, 2018 was initiated via the PubMed (www.ncbi.nlm.nih.gov/pubmed) search engine. The search under “(perceptual priming OR conceptual priming OR repetition priming) AND (fMRI OR functional MRI)” produced a total of 308 relevant studies. The following criteria were used on the selection of these articles for evaluation and analysis (Figure 1):

1. Only studies conducting fMRI on healthy participants were selected.
2. Only studies using implicit, priming tasks, such as perceptual identification, naming, and conceptual judgment (e.g., semantic categorization) were selected. Those using passive viewing, motor learning, and explicit memory tasks were excluded. Negative priming, semantic priming, and masked/subliminal priming were not the focus of this meta-analysis, so studies using them were also excluded.
3. Only studies showing significant behavioral priming were selected. Priming was assessed by a decrease in reaction time, an increase in accuracy, or a bias in response to primed stimulus. When the behavioral results are collected with a separate group of participants different from those in the fMRI experiments, only studies using the same design in both behavioral and fMRI experiments were included.
4. Only studies using visual stimuli were included, such as words or pictures, since visual stimulation has been the dominant modality in fMRI studies. Note that some studies primed the same “item,” but in a different visual format the first vs. second time (e.g., the word “apple” followed by a picture of an apple).
5. To be compatible with ALE approach, only studies showing peak coordinate foci in either Montreal Neurological Institute (MNI) or Talairach space for a whole-brain analysis were included. Those involving region of interest (ROI) analyses were excluded. We only included whole-brain analyses in order to increase the number of the foci and thus the power of each individual meta-analysis.

The above criteria resulted in the selection of 51 studies. Through reviewing the reference lists of the collected papers, additional 14 relevant studies that passed the selection criteria were collected, producing 65 in total.

Contrasts (i.e., experiments) from these studies were extracted for analysis. The contrasts used for identifying RS were New > Old, Novel > Repeated, or First presentation > Second presentation; contrasts for RE were the opposite, i.e., New < Old, Novel < Repeated, or First presentation < Second presentation. To minimize the possibility that meta-analytic results are driven by within-experiment and within-group effects (Turkeltaub et al., 2012), at most one RS contrast and one RE contrast from one experiment within each study was selected. This resulted in 27 studies reporting both RS and RE, 38 studies reporting RS only, and none of them reporting only RE.

Some of these studies demonstrated RS and/or RE in more than one contrast. For instance, the experiment of (Bunzeck et al., 2006) involved two stimulus categories, faces and scenes. Behavioral priming, as well as RS and RE were observed in both categories. Under this circumstance, we collected only the contrasts that involved all stimulus types (e.g., new faces and scenes > old faces and scenes for RS, and old faces and scenes > new faces and scenes for RE). If the studies did not report the results from the contrast All novel > All repeated for RS, or All novel < All repeated for RE, we chose the contrasts from the most typical stimulus type (e.g., neutral faces instead of emotional faces; words instead of pseudowords). However, eight studies included two contrasts for RS and/or RE because the contrasts were from different groups of participants (e.g., young and old; Daselaar et al., 2005; Bergerbest et al., 2009; Ballesteros et al., 2013), different tasks (Schott et al., 2005; Zago et al., 2005), or different types of stimuli (e.g., objects and words; Chouinard and Goodale, 2012; Heusser et al., 2013). Overall, the 65 studies provided data for 100 experiments.

### Data Analysis

Five sets of meta-analyses were performed. The first meta-analysis analyzed all of the priming experiments reporting RS (*n* = 72). Second, the experiments showing RE were analyzed (*n* = 28).

For the third analysis, every experiment was classified as perceptual priming task or conceptual priming task based on the type of task used. Perceptual priming tasks were defined as those involved data-driven processes such as word-stem completion, lexical decision, naming, and perceptual identification (*n* = 27). Conceptual priming tasks were defined as those involved conceptually driven processes including category generation and the conceptual judgment (*n* = 45). Table 1 lists the number and tasks of perceptual and conceptual priming experiments; the supplementary material available online provides a more detailed description of all of the studies and experiments in the meta-analysis.

**Table 1:** Number of experiments and included tasks in perceptual and conceptual priming.

	**RS**	**RE**	**Task**	**Number of experiments**
Perceptual	27	11	Reading/naming/identification	16
			Lexical decision	10
			Perceptual judgment	7
			Word-stem completion	5
Conceptual	45	17	Conceptual judgment	59
			Category generation	3
Total	72	28		

For comparison of conceptual and perceptual priming, we performed both a conjunction analysis (looking for commonalities) and a subtraction analysis (looking for differences in RS between conceptual vs. perceptual priming).

A fourth meta-analysis investigated conceptual priming without perceptual overlap, that is, the stimuli were primed by different object exemplars, views, or segments (*n* = 5). Finally, since picture naming has been proposed to involve both perceptual and conceptual processing (see Discussion), in a fifth analysis, we repeated the conjunction and subtraction analyses but after removing the 6 studies that used picture naming.

### Meta-Analysis Techniques

The software GingerALE 2.3.6 (http://www.brainmap.org/ale) was used to conduct the ALE meta-analyses and the conversion of coordinates reported in MNI space into Talairach space. The ALE meta-analysis is a coordinate-based method capable of determining the brain regions showing an above-chance level of activation convergence across a set of independent studies. In the ALE approach, the foci extracted from the selected studies are not treated as single points, but rather are modeled as the centers of three-dimensional Gaussian probability distribution taking into account spatial uncertainty due to the between-subject and between-template variance (Eickhoff et al., 2009). The width of probability distributions (i.e., full-width at half-maximum, FWHM) is determined by the number of subjects in each experiment. The probabilities of all activation foci in a certain experiment were then combined for each voxel by taking the union, yielding a modeled activation map (MA map). Voxel-wise ALE scores resulted from the union across the MA maps among the experiments that represented the likelihood of activation convergent at particular locations.

To enable spatial inference on these ALE scores, a *p*-value was calculated for each voxel based on the probability of observing an ALE value higher than the value under the null-distribution. This is achieved by randomly relocating ALE values throughout the brain, that is, via random permutation. In this study, the *p*-values were generated by 10,000 permutations. Followed by guidelines recommended by the developers of this method (Eickhoff et al., 2012), the statistical maps were finally thresholded at family-wise error (FWE) corrected *p* < 0.05 with an initial cluster-forming threshold of uncorrected *p* < 0.001 to enforce a minimum cluster size. The threshold of contrast analysis was set at *p* < 0.05, FDR corrected, with permutations = 10,000 and minimum volume = 0 mm^3^. GingerALE employs the term “contributing studies,” to describe studies that are located within the boundaries of ALE cluster. However, it is possible that other studies located near these boundaries but outside of the cluster could have also contributed to it.

The thresholded ALE result images were visualized using the Mango 4.0.1 (San Antonio, TX: UT Health Science Center Research Imaging Institute) and overlaid onto a Talairach-based anatomical template (http://www.brainmap.org/ale/colin_tlrc_2x2x2.nii). Local maxima of activation clusters were anatomically labeled with visual reference to an anatomical atlas provided by Mango. The clusters in the result tables were ordered by region, from frontal, parietal, to occipitotemporal region.

## Results

### Repetition Suppression in All Priming Tasks

**Figure 2:**
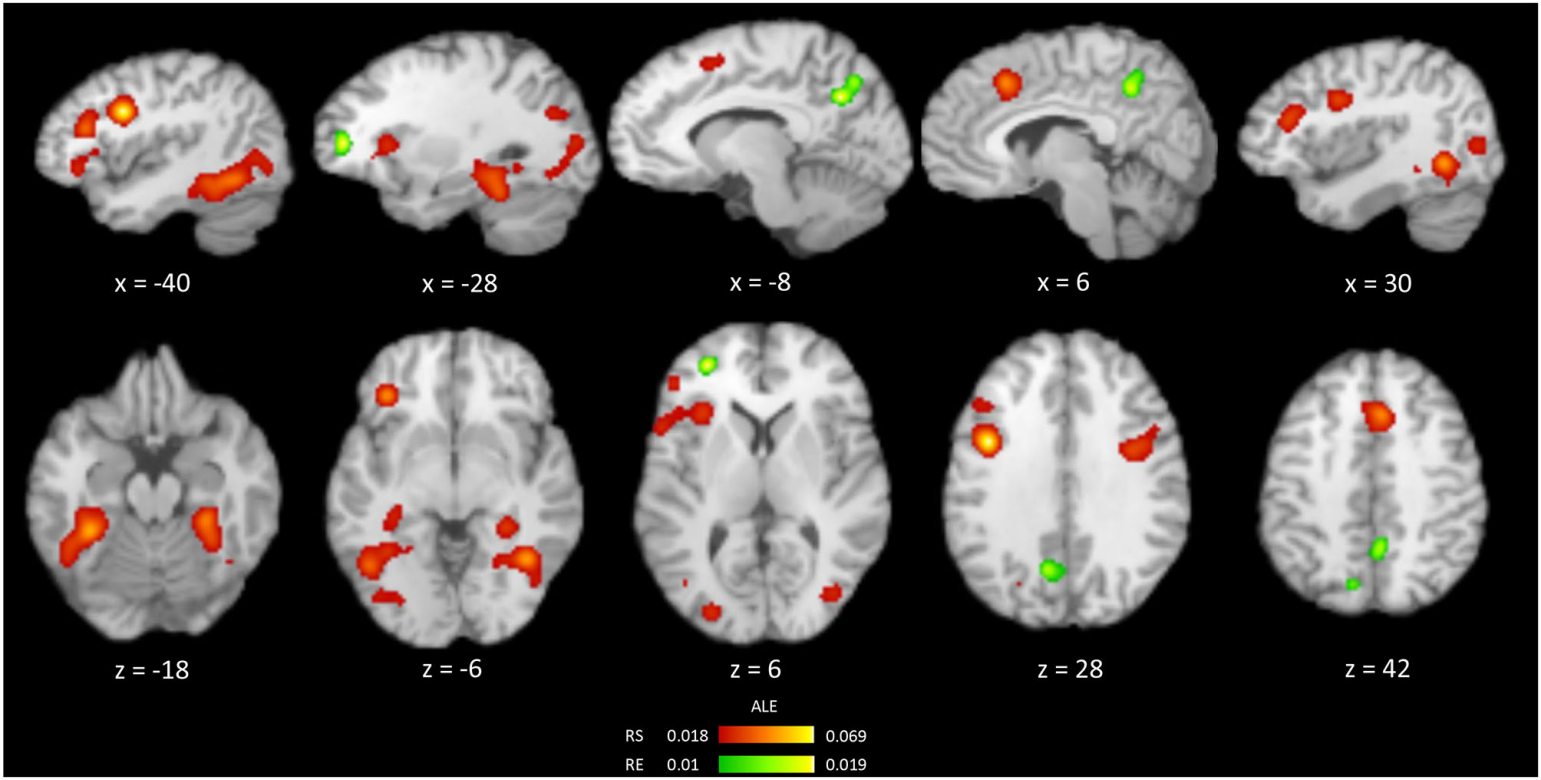
Brain regions showing the occurrences of RS (red) and RE (green) from an ALE meta-analysis of all priming experiments.

Results of the meta-analysis on the occurrences of repetition suppression (RS) from all priming experiments (*n* = 72) are shown in Table 2, Figure 2. These experiments contained 751 activation foci from 1,128 participants. RS was mainly associated with the bilateral fusiform gyrus (FG), inferior frontal gyrus (IFG), and middle occipital gyrus. More specifically, the significant clusters were centered within the bilateral FG, left IFG, right cingulate gyrus, right precentral gyrus, right middle occipital gyrus, and left precuneus.

**Table 2:** Brain regions showing RS or RE across all priming contrasts.

**Cluster no.**	**Cluster size (mm^3^)**	**ALE Value**	**Talairach coordinates**			**Region**	**No. of contributing experiments**	
			**x**	**y**	**z**			**%**
**RS in all priming tasks (*n* = 72)**								
1	6,936	0.069	−40	4	28	L inferior frontal gyrus	26	36.11
		0.046	−46	24	18	L inferior frontal gyrus		
2	4,144	0.048	−34	28	−4	L inferior frontal gyrus	23	31.94
		0.031	−46	36	2	L inferior frontal gyrus		
		0.029	−30	20	6	L insula		
		0.026	−52	12	6	L precentral gyrus		
3	3,960	0.045	6	18	42	R cingulate gyrus	15	20.83
		0.032	−2	14	48	L superior frontal gyrus		
		0.028	−4	4	52	L medial frontal gyrus		
4	2,192	0.035	36	0	30	R precentral gyrus	15	20.83
		0.021	48	10	26	R inferior frontal gyrus		
5	1,568	0.034	42	28	18	R middle frontal gyrus	10	13.89
6	960	0.026	−26	−74	24	L precuneus	7	9.72
7	14,168	0.051	−34	−44	−18	L culmen	47	65.28
		0.047	−44	−60	−10	L fusiform gyrus		
		0.027	−26	−86	6	L middle occipital gyrus		
		0.025	−40	−72	2	L middle occipital gyrus		
		0.023	−24	−72	−12	L Declive		
		0.022	−32	−78	−8	L Inferior Occipital Gyrus		
8	7,200	0.049	40	−58	−6	R fusiform gyrus	31	43.06
		0.046	28	−38	−16	R fusiform gyrus		
		0.024	24	−60	−8	R fusiform gyrus		
		0.023	44	−68	−6	R inferior occipital gyrus		
		0.019	42	−42	−10	R fusiform gyrus		
9	1544	0.027	34	−78	12	R middle occipital gyrus	9	12.50
		0.027	38	−76	4	R middle occipital gyrus		
**RE in all priming tasks (*n* = 28)**								
1	800	0.019	−28	44	6	L middle frontal gyrus	4	14.29
2	1,776	0.019	−8	−64	30	L cuneus	7	25.00
		0.016	−10	−72	40	L precuneus		
3	1,088	0.017	6	−52	38	R precuneus	5	17.86
		0.016	4	−54	44	R precuneus		

### Repetition Enhancement in All Priming Tasks

The meta-analysis of 28 experiments reporting repetition enhancement (RE) contained 158 activation foci from 421 participants. The results showed the significant clusters located in the bilateral precuneus and left middle frontal gyrus (Table 2, Figure 2).

### Repetition Suppression in Perceptual and Conceptual Priming Tasks

**Figure 3:**
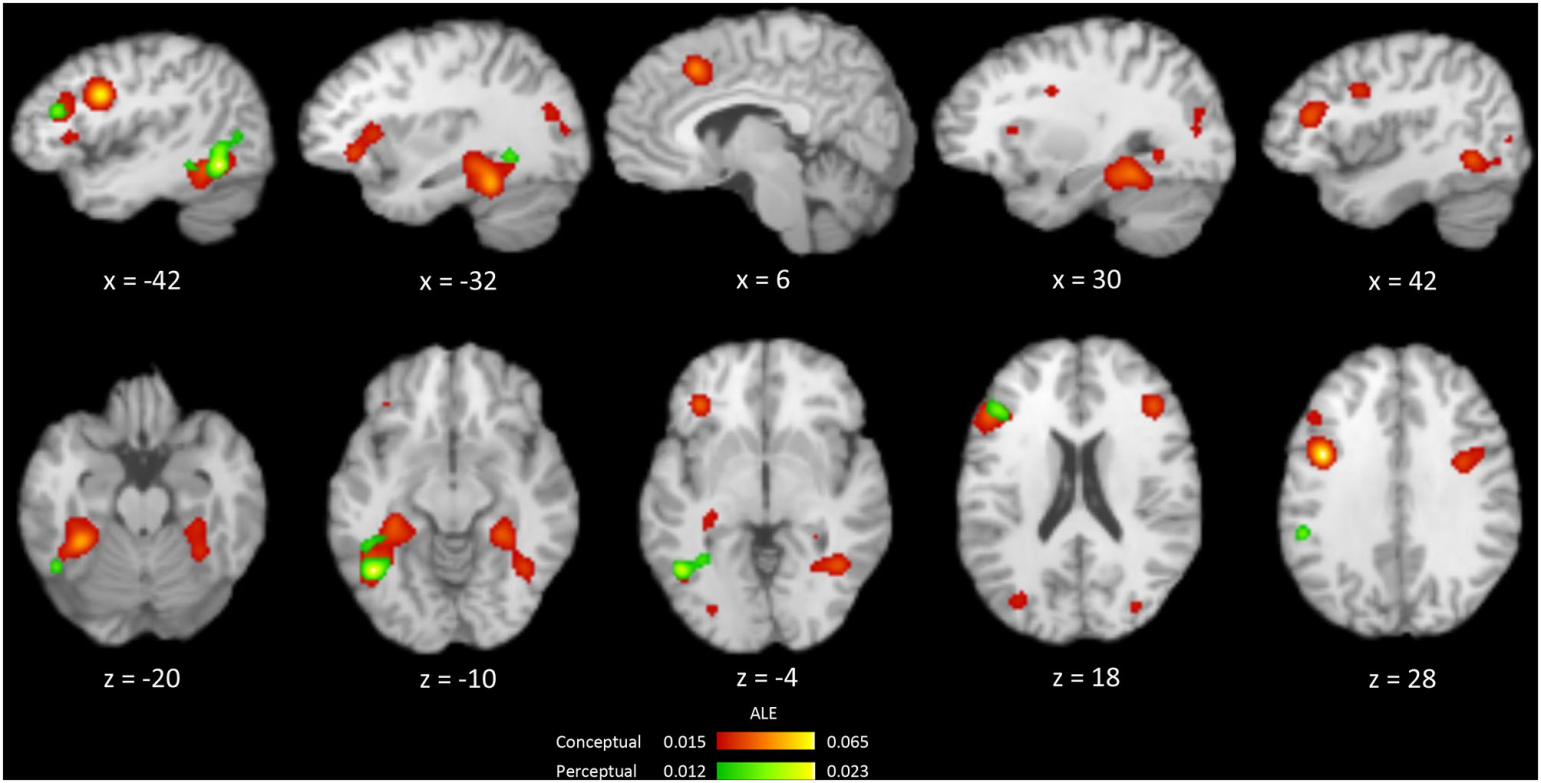
Brain regions showing the occurrences of repetition suppression from an ALE meta-analysis of the conceptual (red) and perceptual (green) priming experiments.

From 263 foci and 422 subjects in 27 perceptual priming experiments, the ALE analysis revealed three significant clusters including the left FG, left middle frontal gyrus and left insula (Table 3, Figure 3). The ALE analysis of 488 foci and 706 subjects in 45 conceptual priming experiments revealed 9 clusters in the bilateral FG, bilateral IFG, bilateral middle frontal gyrus, and bilateral middle occipital gyrus (Figure 3).

**Table 3:** Brain regions showing RS in perceptual and conceptual priming separately.

**Cluster no.**	**Cluster size (mm^3^)**	**ALE Value**	**Talairach coordinates**			**Region**	**No. of contributing experiments**	
			**x**	**y**	**z**			**%**
**RS in perceptual priming tasks (*n* = 27)**								
1	712	0.017	−42	26	18	L middle frontal gyrus	4	14.81
2	672	0.019	−52	−38	24	L insula	5	18.52
3	3,440	0.023	−42	−60	−10	L fusiform gyrus	15	55.56
		0.021	−44	−60	−2	L middle temporal gyrus		
		0.017	−46	−48	−14	L fusiform gyrus		
		0.014	−40	−70	4	L middle occipital gyrus		
**RE in conceptual priming tasks (*n* = 45)**								
1	5,720	0.065	−40	4	28	L inferior frontal gyrus	19	42.22
		0.035	−48	24	20	L inferior frontal gyrus		
2	2,664	0.035	−34	30	−4	L inferior frontal gyrus	14	31.11
		0.026	−30	20	6	L insula		
		0.020	−44	20	6	L inferior frontal gyrus		
3	3,376	0.042	6	18	42	R cingulate gyrus	12	26.67
		0.032	−2	14	48	L superior frontal gyrus		
		0.016	−2	4	52	L superior frontal gyrus		
4	2,320	0.032	42	28	18	R middle frontal gyrus	12	26.67
		0.024	34	20	10	R insula		
		0.019	46	12	22	R inferior frontal gyrus		
5	1,448	0.031	36	0	30	R precentral gyrus	9	20.00
6	8,584	0.048	−32	−44	−20	L culmen	27	60.00
		0.032	−44	−54	−12	L fusiform gyrus		
		0.027	−44	−62	−10	L fusiform gyrus		
7	5,496	0.038	30	−44	−14	R fusiform gyrus	21	46.67
		0.036	40	−58	−6	R fusiform gyrus		
		0.016	42	−70	−8	R inferior occipital gyrus		
8	1,656	0.021	−28	−74	22	L precuneus	11	24.44
		0.021	−34	−76	16	L middle occipital gyrus		
		0.020	−26	−84	2	L middle occipital gyrus		
		0.020	−28	−82	−2	L middle occipital gyrus		
		0.017	−34	−84	10	L middle occipital gyrus		
9	1,424	0.025	34	−78	12	R middle occipital gyrus	7	15.56
		0.021	38	−78	4	R middle occipital gyrus		

**Figure 4:**
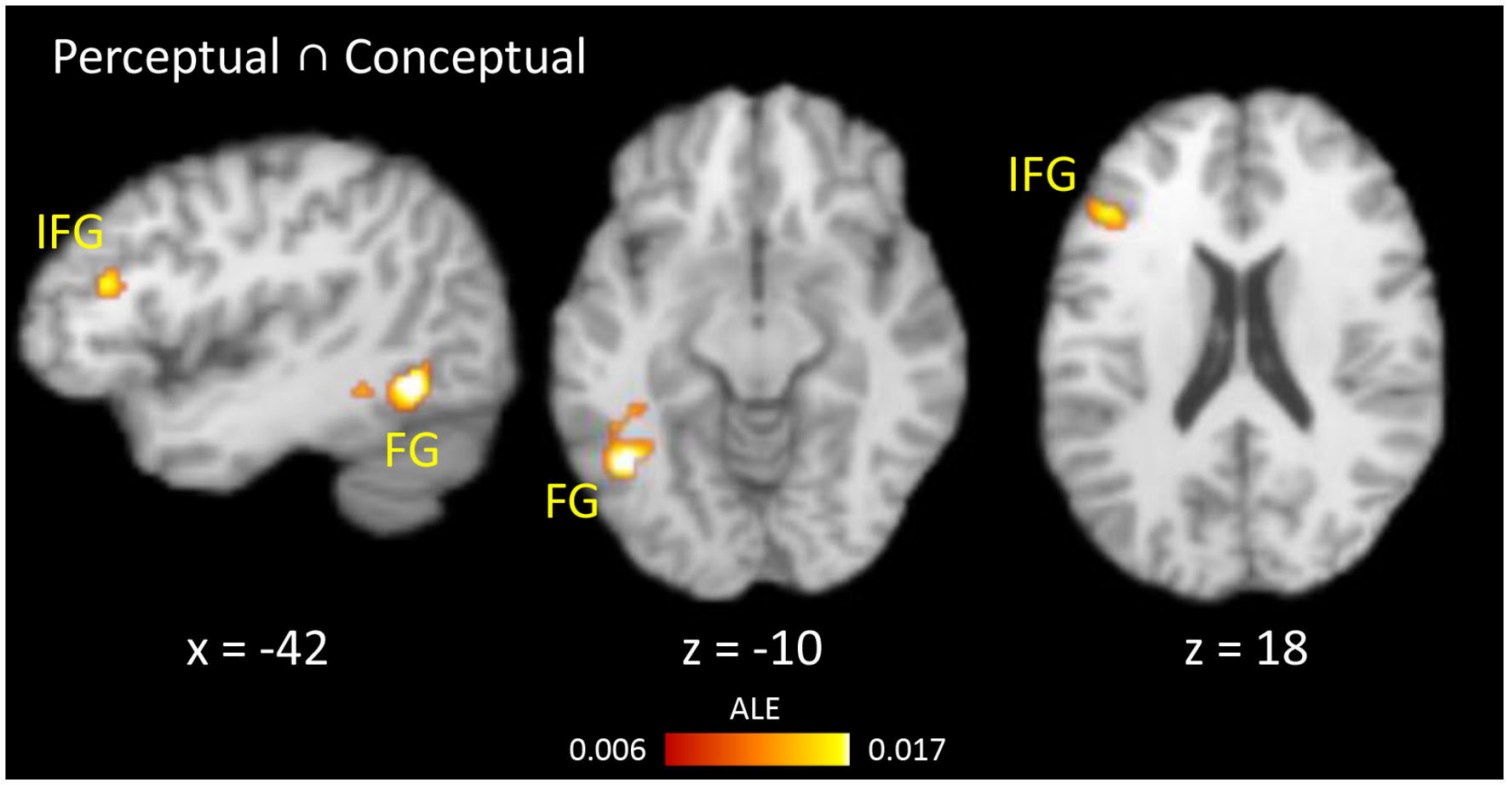
Results of conjunction analysis. Brain regions showing RS for both conceptual and perceptual priming.

Common regions showing RS for both perceptual and conceptual priming were calculated using a conjunction analysis (Figure 4). The overlapping regions were left FG and left IFG. The differences between RS during perceptual and conceptual priming were analyzed by performing subtraction analysis across their thresholded ALE maps. A direct contrast between the two types of priming showed no significant cluster.

### Repetition Suppression in Conceptual Priming Tasks Without Perceptual Overlap

To address whether RS in the fusiform and occipital gyri during conceptual priming tasks is caused by repetition of the same stimulus, in a separate analysis we included only experiments in which primed stimuli were perceptually different from unprimed stimuli (i.e., different exemplars, views, and segments) (*n* = 5). The results of the meta-analysis still showed consistent RS in one area, the left FG.

### Repetition Suppression in Perceptual Priming Tasks Without Picture Naming

Six studies using picture naming task were excluded from perceptual priming because it has been proposed that naming involves conceptual processing as well (see Discussion). The remaining 21 perceptual priming experiments contained 218 activation foci from 347 participants. Only one region, the left FG, was now revealed by the conjunction analysis (i.e., the IFG was no longer present), though still no region showed an interaction between RS and perceptual/conceptual priming in the subtraction analysis.

## Discussion

The present study performed meta-analyses across 65 studies that used fMRI to study the neural correlates of behavioral repetition priming. When including all studies, repetition suppression (RS) was consistently observed in bilateral inferior frontal gyrus (IFG) and fusiform gyrus (FG); whereas repetition enhancement (RE) was consistently shown in bilateral precuneus and middle frontal gyrus. Interestingly, a conjunction analysis showed that RS occurred in left IFG and left FG during both perceptual and conceptual priming tasks, and a subtraction analysis provided no evidence for any region implicated in one task but not the other. In other words, there was little evidence for a qualitative dissociation between the neural correlates of perceptual and conceptual priming.

### RS in Fusiform Gyrus and Inferior Frontal Gyrus

The prevalence of RS in FG and IFG is consistent with the previous meta-analyses on RS (Kim, 2016, 2019), which included many of the same studies, but did not restrict analysis to those studies that showed concurrent behavioral priming. However, our results did not show RS in hippocampus, which is believed to reflect priming of relational processing of information (Kim, 2019), most likely because most of the studies here used single-item priming. Given the debate about whether hippocampus is only involved in explicit memory, or can also be involved in implicit relational memory (e.g., Henke, 2010), more neuroimaging studies are needed on implicit relational priming.

Previous neuropsychological and neuroimaging studies on priming have suggested that the occipitotemporal and frontal regions are the critical areas for mediating perceptual and conceptual priming, respectively (Wiggs and Martin, 1998; Schacter et al., 2007; Gong et al., 2016). The component process view proposes that RS reflects the facilitation of processes that are engaged during both initial and primed presentation of a stimulus, and therefore occurs in brain regions specialized for those processes (Schacter and Buckner, 1998; Henson, 2003). For instance, RS in occipitotemporal regions (such as fusiform cortex) is thought to reflect the facilitation of visual perceptual processes (Blaxton, 1999; Henson, 2003), whereas RS in prefrontal regions (such as inferior frontal gyrus) is thought to reflect the facilitation of conceptual processes (Wagner et al., 1997, 2000). However, our meta-analysis provided less support for this putative division of labor, since we found that FG showed RS for both perceptual and conceptual priming, and IFG showed RS for conceptual and/or perceptual priming.

Prima facie, this is inconsistent with the component process view. However, it is possible that the studies we assigned to examples of perceptual vs. conceptual priming were not “process pure,” in the sense that both perceptual and conceptual processes might have been facilitated, even if the stimuli and task were designed to emphasize one type of process over the other. This ambiguity might have led to the wrong contrasts/studies being assigned to the wrong category of conceptual vs. perceptual in this meta-analysis, and might even explain why a clear dissociation was not found between these two types of priming. An example of this would be in studies of conceptual priming that use a semantic task (assumed to engage conceptual processes) but also repeat the stimulus in the same format (e.g., words), which is likely to result in concurrent facilitation of perceptual processes too. However, when we performed a subsidiary analysis of just those studies that changed the (visual) format of the initial and primed presentation of an item, there was still evidence of RS in FG (though it is worth noting that the number for this analysis of “pure” conceptual priming is below the number of 17–20 experiments suggested by Eickhoff et al., 2016). While it is possible that FG is involved in conceptual processes as well as IFG, this finding questions the neuroimaging support for the neural dissociation between conceptual and perceptual priming (e.g., from neuropsychological data).

We also found RS in IFG for perceptual priming, which is discussed even less in the literature, and not easy to explain by the component process view. However, another debate concerns studies using picture naming. Picture naming was included as an example of perceptual processing in early studies (Wiggs and Martin, 1998). Subsequent research however, particularly using bilingual participants, suggests that picture naming can be decomposed into object identification and word production: object identification is assumed to be a perceptual process, whereas word production requires conceptual access (Francis and Sáenz, 2007; Francis et al., 2008; for review, see Francis, 2020). Factor analysis has also suggested that picture naming taps both perceptual and conceptual factors (Bruss and Mitchell, 2009). We therefore performed a further sub-analysis after having removed studies using picture naming. While there was still no region showing a significant difference in RS between perceptual and conceptual priming in the subtraction analysis, the conjunction analysis now revealed a common RS only in FG. Thus, we cannot make strong conclusions about the role of IFG in perceptual priming, if naming tasks are excluded as an example of perceptual priming: it could be that IFG is only involved in conceptual priming, or the lack of IFG in our sub-analysis could be a false negative owing to the reduced sample size. Further studies are needed to resolve this question, and dissociate the perceptual vs. conceptual contributions to naming.

An alternative interpretation of our findings is that RS in IFG and FG reflects the retrieval of S-R bindings, cued by the repetition of a stimulus, where this rapid and direct retrieval of a response bypasses the need for “re-running” the component processes involved on first presentation of the stimulus, leading to RS (Dobbins et al., 2004; for review, see Henson et al., 2014). There is compelling evidence that S-R bindings make a major contribution to behavioral priming in the speeded classification tasks that were used in the majority of priming tasks considered here, and such bindings can include multiple, abstract representations of stimuli, and responses, which would allow them to generalize across visual format for example (e.g., Horner and Henson, 2009, 2011). It seems likely that IFG in particular is involved in control processes like response generation, and therefore prone to showing the facilitatory effect of retrieving a response directly from an S-R binding in either perceptual or conceptual priming. This might explain why the most common region to show correlations between RS and behavioral priming in previous studies is IFG. The role of FG in retrieving S-R bindings is less clear, particularly when residual RS in FG has been found despite minimizing the contribution of S-R bindings (Horner and Henson, 2008; Race et al., 2008), and when there have been few reports that FG RS correlates with behavioral priming. It is possible that facilitation of perceptual processes (according to component process view) does occur in FG, but this explains only a minor proportion of the variance in behavioral priming (compared that attributed to decision processes in IFG). This hybrid account (perceptual facilitation in FG and S-R retrieval in IFG) is supported by ERP studies that suggest that repetition effects in FG are time-locked to stimulus onset, while repetition effects in IFG are time-locked to response onset (Race et al., 2008; Horner and Henson, 2011; Wig, 2012).

One way to distinguish the component process account from the S-R binding account is to manipulate the task (response requirements) so that S-R bindings become unhelpful, potentially revealing only the underlying component processes (see Henson et al., 2014). Indeed, when reversing the task between initial and primed presentations, such that the responses retrieved from S-R bindings conflict with those generated by component processes, IFG can even show RE rather than RS (Horner and Henson, 2011), suggesting that the S-R binding account explains the IFG findings better than the component process account. Unfortunately there were insufficient numbers of studies for the current meta-analysis to compare tasks where the same response was repeated (congruent) vs. altered or reversed (incongruent), which would help interpret the role of RS in the two key areas of IFG and FG identified here. Another potential approach is to use techniques like transcranial magnetic stimulation (TMS) to directly modulate the processing in certain brain regions. For example, Wig et al. (2005) applied TMS to IFG and found that both RS in this region, and behavioral priming, were reduced in a visual object semantic classification task. While this could be explained by disruption of conceptual processing or retrieval of S-R bindings, it is interesting that RS in a posterior visual region (middle occipital gyrus, though not FG) was not affected by the TMS to IFG, despite the reduction in behavioral priming. This is consistent with the above hybrid account, where RS in those regions is related to perceptual facilitation, but this facilitation makes negligible contribution to behavioral priming (relative to conceptual facilitation) in semantic tasks like this. Further evidence to distinguish these possibilities requires TMS to perceptual regions like FG.

### RS in Other Regions

Like Wig et al. (2005), we did find RS in middle occipital gyrus when considering all priming experiments, but not in conceptual priming tasks without perceptual overlap, suggesting that this region is involved in relatively low-level visual processing. Our results also showed RS in precentral gyrus in all experiments. This could be related to priming of motor responses, but we think this is unlikely, because in most studies reviewed, the specific motor response was orthogonal to the contrast of primed vs. unprimed trials (e.g., left and right index fingers were pressed equally often for both primed and unprimed trials) and few studies involved immediate repetition of the same stimulus and response.

### RE in Precuneus and Middle Frontal Gyrus

Only two regions showed RE in the current priming tasks: precuneus and middle frontal gyrus. Precuneus is a region that often shows activation for primed vs. initial stimuli in explicit memory tasks, and has been associated with recollection and visual imagery in particular. It seems likely that the RE found in the present implicit tasks reflects incidental (possibly involuntary) recollection of the initial presentation of a stimulus, even though such recollection is not needed to perform the priming task (Donaldson et al., 2001; Henson et al., 2002; Kim and Yassa, 2013; Gomes et al., 2016; Poppenk et al., 2016; see also Kim, 2016). An alternative explanation is that precuneus responds more to easier tasks, e.g., more to primed than novel objects (Korsnes et al., 2008; Korsnes and Magnussen, 2014). It is also possible that regions showing RE, such as precuneus, are “task-negative” regions. Such task-negative regions might receive less suppressive input from other “task-positive” regions, leading to RE. The role of RE in middle frontal gyrus is less clear: it could also reflect incidental explicit memory, though this region is often associated more with monitoring the products of explicit memory (Henson et al., 2000) and a typical implicit task would not seem require such post-retrieval monitoring, unless it reflects decision processes related to the use of S-R bindings vs. component processes (see above).

Interestingly, none of the regions typically associated with priming (e.g., from neuropsychology) showed evidence of RE (rather than RS). This may be because the majority of studies in the present meta-analysis used pre-experimentally familiar rather than unfamiliar stimuli (see Henson et al., 2000; Segaert et al., 2013; Makukhin and Bolland, 2014). Another possibility is that distinct populations of neurons exhibit suppressed vs. enhanced responses to primed stimuli (de Gardelle et al., 2013), so that the overall response of a brain region (or voxel) depends on the ratio of these neuronal populations. Further studies are needed to investigate when priming is associated with RE vs. RS, but what is clear is that the association of RS with implicit memory and RE with explicit memory is likely to be too simplistic (e.g., Henson, 2003).

### Limitation and Future Directions

The present study possesses inherent limitations of the meta-analysis approach. First is the most important issue about which experiments or contrasts are included in the meta-analysis. For example, we included studies in which stimuli repeat either with or without intervening trials. However, the neural mechanism of immediate priming (or fMRI adaptation; Grill-Spector and Malach, 2001) may differ from priming over longer delays (e.g., Epstein et al., 2008). Other factors that might affect the distribution and direction of repetition effects include attention (Eger, 2004; Yi, 2005), familiarity (Henson et al., 2000; Korsnes et al., 2008; Soldan et al., 2008, 2010) and number of repetitions (Müller et al., 2013). We included all these studies in order to increase the sensitivity of our meta-analysis to the regions generally associated with priming. A second limitation is that, while our conjunction analyses provided positive evidence for shared RS in IFG and FG for conceptual and perceptual priming, the lack of other regions showing a significant difference in RS for conceptual and perceptual priming is a null result that cannot be used to argue that there are no differences. A third limitation is that ALE calculations do not consider the size of an effect (only where it is significant). Thus if conceptual and perceptual priming both produced significant RS in the IFG and FG, but to differing degrees (e.g., greater RS in IFG for conceptual than perceptual priming, and greater RS in FG for perceptual than conceptual priming), then this would not be apparent in the current type of meta-analysis.

Our results also suggest some possible directions for future studies. Firstly, Schacter et al. (2007) stated that there is scant evidence of correlation between RS in posterior regions and behavioral priming effects. Indeed, there has not been strong evidence on the correlation of behavioral perceptual priming and RS in brain regions such as occipital and temporal lobe. One possibility is that the type of correlation analysis is not appropriate: Previous studies measured Pearson's correlation between the magnitude of priming and RS across subjects. However, the measurements of correlations across trials within subject may provide greater sensitivity and avoid other sources of inter-subject variability.

A second important issue is how IFG communicates with occipitotemporal gyrus. Some theoretical models such as synchronization (Gotts et al., 2012) and predictive coding (Grotheer and Kovács, 2016) emphasize the interaction between different regions. If so, changes in between-region functional and effective connectivity should be identified, in addition to changes in regional activity. For instance, dynamic causal modeling (DCM) suggested that repetition of body images is associated with changes in backward (fusiform body area, FBA, to extrastriate body area, EBA), as well as forward, (EBA-to-FBA), connections (Ewbank et al., 2011).

Finally, it is surprising that we did not find brain correlates of conceptual priming in the anterior temporal lobe (ATL) or in perirhinal cortex, which are both regions that previous lesion and imaging studies have implicated in semantic processing (Wright et al., 2015; Ralph et al., 2017). One reason might be the fMRI signal loss in these regions when using conventional gradient-echo fMRI acquisition, and future studies could employ methods to minimize this signal loss.

## Conclusion

The present meta-analyses identify RS in fusiform gyrus and inferior frontal gyrus, and RE in precuneus and middle frontal gyrus, during behavioral priming tasks. The overlap in regions exhibiting RS across perceptual and conceptual priming is not predicted by previous studies and reviews, nor expected based on human lesion data. One alternative to the component process view is that the inferior frontal RS reflects the retrieval of S-R bindings, and the fusiform RS reflects the facilitated perceptual/conceptual processing of the stimuli. Thus, future studies should attempt to examine how S-R bindings and component processes interact to determine the magnitude of RS and priming.

## Data Availability Statement

All datasets presented in this study are included in the article/Supplementary Material.

## Author Contributions

S-ML performed the analysis and drafted the manuscript. RH and C-YL revised it. All authors contributed to the conception of work and interpretation of data. All authors contributed to the article and approved the submitted version.

## Conflict of Interest

The authors declare that the research was conducted in the absence of any commercial or financial relationships that could be construed as a potential conflict of interest.

## References

[B1] Ballesteros S. Bischof G. N. Goh J. O. Park D. C. (2013). Neural correlates of conceptual object priming in young and older adults: an event-related functional magnetic resonance imaging study. Neurobiol. Aging 34, 1254–1264. 10.1016/j.neurobiolaging.2012.09.019 23102512 PMC4028122

[B2] Bergerbest D. Gabrieli J. D. E. Whitfield-Gabrieli S. Kim H. Stebbins G. T. Bennett D. A.. (2009). Age-associated reduction of asymmetry in prefrontal function and preservation of conceptual repetition priming. Neuroimage 45, 237–246. 10.1016/j.neuroimage.2008.10.019 19015038 PMC2761100

[B3] Blaxton T. A. (1989). Investigating dissociations among memory measures: Support for a transfer-appropriate processing framework. J. Exp. Psychol. 15, 657–668. 10.1037/0278-7393.15.4.657

[B4] Blaxton T. A. (1999). Combining disruption and activation techniques to map conceptual and perceptual memory processes in the human brain, in Memory: Structure, Function, or Process? eds Foster J. K. Jelicic M. (Oxford: Oxford University Press), 104–129.

[B5] Brooks S. J. Savov V. Allzén E. Benedict C. Fredriksson R. Schiöth H. B. (2012). Exposure to subliminal arousing stimuli induces robust activation in the amygdala, hippocampus, anterior cingulate, insular cortex and primary visual cortex: a systematic meta-analysis of fMRI studies. Neuroimage 59, 2962–2973. 10.1016/j.neuroimage.2011.09.077 22001789

[B6] Bruss P. J. Mitchell D. B. (2009). Memory systems, processes, and tasks: taxonomic clarification via factor analysis. Am. J. Psychol. 122, 175–189. Retrieved from: http://www.jstor.org/stable/27784390 19507425

[B7] Buckner R. L. Petersen S. E. Ojemann J. G. Miezin F. M. Squire L. R. Raichle M. E. (1995). Functional anatomical studies of explicit and implicit memory retrieval tasks. J. Neurosci. 15, 12–29. 10.1523/JNEUROSCI.15-01-00012.1995 7823123 PMC6578325

[B8] Bunzeck N. Schütze H. Düzel E. (2006). Category-specific organization of prefrontal response-facilitation during priming. Neuropsychologia 44, 1765–1776. 10.1016/j.neuropsychologia.2006.03.019 16701731

[B9] Chouinard P. A. Goodale M. A. (2012). FMRI-adaptation to highly-rendered color photographs of animals and manipulable artifacts during a classification task. Neuroimage 59, 2941–2951. 10.1016/j.neuroimage.2011.09.073 22015854

[B10] Daselaar S. M. Veltman D. J. Rombouts S. A. R. B. Raaijmakers J. G. W. Jonker C. (2005). Aging affects both perceptual and lexical/semantic components of word stem priming: an event-related fMRI study. Neurobiol. Learn. Mem. 83, 251–262. 10.1016/j.nlm.2005.01.005 15820861

[B11] de Gardelle V. Stokes M. Johnen V. M. Wyart V. Summerfield C. (2013). Overlapping multivoxel patterns for two levels of visual expectation. Front. Hum. Neurosci. 7:158. 10.3389/fnhum.2013.00158 23630488 PMC3635018

[B12] Dobbins I. G. Schnyer D. M. Verfaellie M. Schacter D. L. (2004). Cortical activity reductions during repetition priming can result from rapid response learning. Nature 428, 316–319. 10.1038/nature02400 14990968

[B13] Dolan R. J. Fink G. R. Rolls E. Booth M. Holmes A. Frackowiak R. S. J. (1997). How the brain learns to see objects and faces in an impoverished context. Nature 389, 596–599. 10.1038/39309 9335498

[B14] Donaldson D. I. Petersen S. E. Buckner R. L. (2001). Dissociating memory retrieval processes using fMRI. Neuron 31, 1047–1059. 10.1016/S0896-6273(01)00429-9 11580903

[B15] Eger E. (2004). BOLD repetition decreases in object-responsive ventral visual areas depend on spatial attention. J. Neurophysiol. 92, 1241–1247. 10.1152/jn.00206.2004 15056686

[B16] Eickhoff S. B. Bzdok D. Laird A. R. Kurth F. Fox P. T. (2012). Activation likelihood estimation meta-analysis revisited. Neuroimage 59, 2349–2361. 10.1016/j.neuroimage.2011.09.017 21963913 PMC3254820

[B17] Eickhoff S. B. Laird A. R. Grefkes C. Wang L. E. Zilles K. Fox P. T. (2009). Coordinate-based activation likelihood estimation meta-analysis of neuroimaging data: a random-effects approach based on empirical estimates of spatial uncertainty. Hum. Brain Mapp. 30, 2907–2926. 10.1002/hbm.20718 19172646 PMC2872071

[B18] Eickhoff S. B. Nichols T. E. Laird A. R. Hoffstaedter F. Amunts K. Fox P. T.. (2016). Behavior, sensitivity, and power of activation likelihood estimation characterized by massive empirical simulation. Neuroimage 137, 70–85. 10.1016/j.neuroimage.2016.04.072 27179606 PMC4981641

[B19] Epstein R. A. Parker W. E. Feiler A. M. (2008). Two kinds of fMRI repetition suppression? evidence for dissociable neural mechanisms. J. Neurophysiol. 99, 2877–2886. 10.1152/jn.90376.2008 18400954

[B20] Ewbank M. P. Lawson R. P. Henson R. N. Rowe J. B. Passamonti L. Calder A. J. (2011). Changes in “Top-Down” connectivity underlie repetition suppression in the ventral visual pathway. J. Neurosci. 31, 5635–5642. 10.1523/JNEUROSCI.5013-10.2011 21490204 PMC3759805

[B21] Fleischman D. A. (2007). Repetition priming in aging and alzheimer's disease: an integrative review and future directions. Cortex 43, 889–897. 10.1016/S0010-9452(08)70688-9 17941347

[B22] Fleischman D. A. E D. Reminger S. Rinaldi J. Morrell F. Wilson R. (1995). Conceptual priming in perceptual identification for patients with Alzheimer's disease and a patient with right occipital lobectomy. Neuropsychology 9, 187–197. 10.1037/0894-4105.9.2.187

[B23] Fleischman D. A. Gabrieli J. D. E. (1998). Repetition priming in normal aging and Alzheimer's disease: a review of findings and theories. Psychol. Aging 13, 88–119. 10.1037/0882-7974.13.1.88 9533193

[B24] Fleischman D. A. Wilson R. S. Gabrieli J. D. E. Schneider J. A. Bienias J. L. Bennett D. A. (2005). Implicit memory and Alzheimer's disease neuropathology. Brain 128, 2006–2015. 10.1093/brain/awh559 15975947

[B25] Francis W. S. (2020). Shared core meanings and shared associations in bilingual semantic memory: evidence from research on implicit memory. Int. J. Bilingualism 24, 464–477. 10.1177/1367006918814375

[B26] Francis W. S. Corral N. I. Jones M. L. Sáenz S. P. (2008). Decomposition of repetition priming components in picture naming. J. Exp. Psychol. Gen. 137, 566–590. 10.1037/0096-3445.137.3.566 18729716

[B27] Francis W. S. Sáenz S. P. (2007). Repetition priming endurance in picture naming and translation: contributions of component processes. Mem. Cogn. 35, 481–493. 10.3758/BF03193288 17691147

[B28] Gabrieli J. D. E. Fleischman D. A. Keane M. M. Reminger S. L. Morrell F. (1995). Double dissociation between memory systems underlying explicit and implicit memory in the human brain. Psychol. Sci. 6, 76–82. 10.1111/j.1467-9280.1995.tb00310.x

[B29] Gabrieli J. D. E. Vaidya C. J. Stone M. Francis W. S. Thompson-Schill S. L. Fleischman D. A.. (1999). Convergent behavioral and neuropsychological evidence for a distinction between identification and production forms of repetition priming. J. Exp. Psychol. Gen. 128, 479–498. 10.1037/0096-3445.128.4.479 10650584

[B30] George N. Dolan R. J. Fink G. R. Baylis G. C. Russell C. Driver J. (1999). Contrast polarity and face recognition in the human fusiform gyrus. Nat. Neurosci. 2, 574–580. 10.1038/9230 10448224

[B31] Gomes C. A. Figueiredo P. Mayes A. (2016). Priming for novel object associations: neural differences from object item priming and equivalent forms of recognition. Hippocampus 26, 472–491. 10.1002/hipo.22537 26418396

[B32] Gong L. Wang J. Yang X. Feng L. Li X. Gu C.. (2016). Dissociation between conceptual and perceptual implicit memory: evidence from patients with frontal and occipital lobe lesions. Front. Hum. Neurosci. 9:722. 10.3389/fnhum.2015.00722 26793093 PMC4711335

[B33] Gotts S. J. Chow C. C. Martin A. (2012). Repetition priming and repetition suppression: Multiple mechanisms in need of testing. Cogn. Neurosci. 3, 250–259. 10.1080/17588928.2012.697054 24171755 PMC6454549

[B34] Grill-Spector K. Henson R. Martin A. (2006). Repetition and the brain: neural models of stimulus-specific effects. Trends Cogn. Sci. 10, 14–23. 10.1016/j.tics.2005.11.006 16321563

[B35] Grill-Spector K. Malach R. (2001). fMR-adaptation: A tool for studying the functional properties of human cortical neurons. Acta Psychol. 107, 293–321. 10.1016/S0001-6918(01)00019-1 11388140

[B36] Grosse D. A. Wilson R. S. Fox J. H. (1990). Preserved word-stem-completion priming of semantically encoded information in Alzheimer's disease. Psychol. Aging 5, 304–306. 10.1037/0882-7974.5.2.304 2378696

[B37] Grotheer M. Kovács G. (2016). Can predictive coding explain repetition suppression? Cortex 80, 113–124. 10.1016/j.cortex.2015.11.027 26861559

[B38] Henke K. (2010). A model for memory systems based on processing modes rather than consciousness. Nat. Rev. Neurosci. 11, 523–532. 10.1038/nrn2850 20531422

[B39] Henson R. N. (2003). Neuroimaging studies of priming. Prog. Neurobiol. 70, 53–81. 10.1016/S0301-0082(03)00086-8 12927334

[B40] Henson R. N. Eckstein D. Waszak F. Frings C. Horner A. J. (2014). Stimulus–response bindings in priming. Trends Cogn. Sci. 18, 376–384. 10.1016/j.tics.2014.03.004 24768034 PMC4074350

[B41] Henson R. N. Shallice T. Dolan R. (2000). Neuroimaging evidence for dissociable forms of repetition priming. Science 287, 1269–1272. 10.1126/science.287.5456.1269 10678834

[B42] Henson R. N. Shallice T. Gorno-Tempini M. L. Dolan R. J. (2002). Face repetition effects in implicit and explicit memory tests as measured by fMRI. Cereb. Cortex 12, 178–186. 10.1093/cercor/12.2.178 11739265

[B43] Heusser A. C. Awipi T. Davachi L. (2013). The ups and downs of repetition: modulation of the perirhinal cortex by conceptual repetition predicts priming and long-term memory. Neuropsychologia 51, 2333–2343. 10.1016/j.neuropsychologia.2013.04.018 23651708 PMC3902137

[B44] Horner A. J. Henson R. N. (2008). Priming, response learning and repetition suppression. Neuropsychologia 46, 1979–1991. 10.1016/j.neuropsychologia.2008.01.018 18328508 PMC2430995

[B45] Horner A. J. Henson R. N. (2009). Bindings between stimuli and multiple response codes dominate long-lag repetition priming in speeded classification tasks. J. Exp. Psychol. 35, 757–779. 10.1037/a0015262 19379048

[B46] Horner A. J. Henson R. N. (2011). Incongruent abstract stimulus–response bindings result in response interference: FMRI and EEG evidence from visual object classification priming. J. Cogn. Neurosci. 24, 760–773. 10.1162/jocn_a_00163 22066586 PMC3601413

[B47] Jacoby L. L. (1983). Remembering the data: Analyzing interactive processes in reading. J. Verb. Learn. Verb. Behav. 22, 485–508. 10.1016/S0022-5371(83)90301-8

[B48] Keane M. M. Gabrieli J. D. E. Mapstone H. C. Johnson K. A. Corkin S. (1995). Double dissociation of memory capacities after bilateral occipital-lobe or medial temporal-lobe lesions. Brain 118, 1129–1148. 10.1093/brain/118.5.1129 7496775

[B49] Kim H. (2016). Brain regions that show repetition suppression and enhancement: A meta-analysis of 137 neuroimaging experiments. Hum. Brain Mapp. 38, 1894–1913. 10.1002/hbm.23492 28009076 PMC6866918

[B50] Kim H. (2019). Neural correlates of explicit and implicit memory at encoding and retrieval: A unified framework and meta-analysis of functional neuroimaging studies. Biol. Psychol. 145, 96–111. 10.1016/j.biopsycho.2019.04.006 31034858

[B51] Kim J. Yassa M. A. (2013). Assessing recollection and familiarity of similar lures in a behavioral pattern separation task. Hippocampus 23, 287–294. 10.1002/hipo.22087 23401187 PMC4172605

[B52] Korsnes M. S. Magnussen S. J. (2014). FMRI evidence for dissociation between priming and conscious recognition. J. Integr. Neurosci. 13, 509–517. 10.1142/S0219635214500149 25164357

[B53] Korsnes M. S. Wright A. A. Gabrieli J. D. E. (2008). An fMRI analysis of object priming and workload in the precuneus complex. Neuropsychologia 46, 1454–1462. 10.1016/j.neuropsychologia.2007.12.028 18304592

[B54] Lustig C. Buckner R. L. (2004). Preserved neural correlates of priming in old age and dementia. Neuron 42, 865–875. 10.1016/j.neuron.2004.04.002 15182724

[B55] Maccotta L. Buckner R. L. (2004). Evidence for neural effects of repetition that directly correlate with behavioral priming. J. Cogn. Neurosci. 16, 1625–1632. 10.1162/0898929042568451 15601524

[B56] Makukhin K. Bolland S. (2014). Dissociable forms of repetition priming: a computational model. Neural Comput. 26, 712–738. 10.1162/NECO_a_00569 24479780

[B57] Meiran N. Jelicic M. (1995). Implicit memory in Alzheimer's disease: a meta-analysis. Neuropsychology 9, 291–303. 10.1037/0894-4105.9.3.291

[B58] Meneguzzo P. Tsakiris M. Schioth H. B. Stein D. J. Brooks S. J. (2014). Subliminal versus supraliminal stimuli activate neural responses in anterior cingulate cortex, fusiform gyrus and insula: a meta-analysis of fMRI studies. BMC Psychol. 2:52. 10.1186/s40359-014-0052-1 25593703 PMC4271330

[B59] Moher D. Liberati A. Tetzlaff J. Altman D. G. The PRISMA Group (2009). Preferred reporting items for systematic reviews and meta-analyses: the PRISMA statement. PLoS Med. 6:e1000097 10.1371/journal.pmed.1000097 19621072 PMC2707599

[B60] Monti L. A. Gabrieli J. D. E. Reminger S. L. Rinaldi J. A. Wilson R. S. Fleischman D. A. (1996). Differential effects of aging and Alzheimer's disease on conceptual implicit and explicit memory. Neuropsychology 10, 101–112. 10.1037/0894-4105.10.1.101

[B61] Müller N. G. Strumpf H. Scholz M. Baier B. Melloni L. (2013). Repetition suppression versus enhancement—it's quantity that matters. Cereb. Cortex 23, 315–322. 10.1093/cercor/bhs009 22314047

[B62] Poppenk J. McIntosh A. R. Moscovitch M. (2016). fMRI evidence of equivalent neural suppression by repetition and prior knowledge. Neuropsychologia 90, 159–169. 10.1016/j.neuropsychologia.2016.06.034 27461077

[B63] Race E. A. Shanker S. Wagner A. D. (2008). Neural priming in human frontal cortex: multiple forms of learning reduce demands on the prefrontal executive system. J. Cogn. Neurosci. 21, 1766–1781. 10.1162/jocn.2009.21132 18823245 PMC2788302

[B64] Ralph M. A. L. Jefferies E. Patterson K. Rogers T. T. (2017). The neural and computational bases of semantic cognition. Nat. Rev. Neurosci. 18, 42–55. 10.1038/nrn.2016.150 27881854

[B65] Rodd J. M. Vitello S. Woollams A. M. Adank P. (2015). Localising semantic and syntactic processing in spoken and written language comprehension: an activation likelihood estimation meta-analysis. Brain Lang. 141, 89–102. 10.1016/j.bandl.2014.11.012 25576690

[B66] Roediger H. L. McDermott K. B. (1993). Implicit memory in normal subjects. Handb. Neuropsychol. 8, 63–131.

[B67] Salmon D. P. Shimamura A. P. Butters N. Smith S. (1988). Lexical and semantic priming deficits in patients with alzheimer's disease. J. Clin. Exp. Neuropsychol. 10, 477–494. 10.1080/01688638808408254 2969917

[B68] Schacter D. L. Buckner R. L. (1998). Priming and the brain. Neuron 20, 185–195. 10.1016/S0896-6273(00)80448-1 9491981

[B69] Schacter D. L. Reiman E. Uecker A. Roister M. R. Yun L. S. Cooper L. A. (1995). Brain regions associated with retrieval of structurally coherent visual information. Nature 376, 587–590. 10.1038/376587a0 7637806

[B70] Schacter D. L. Wig G. S. Stevens W. D. (2007). Reductions in cortical activity during priming. Curr. Opin. Neurobiol. 17, 171–176. 10.1016/j.conb.2007.02.001 17303410

[B71] Schott B. H. Henson R. N. Richardson-Klavehn A. Becker C. Thoma V. Heinze H.-J.. (2005). Redefining implicit and explicit memory: The functional neuroanatomy of priming, remembering, and control of retrieval. Proc. Natl. Acad. Sci. U.S.A. 102, 1257–1262. 10.1073/pnas.0409070102 15657126 PMC545864

[B72] Segaert K. Weber K. de Lange F. P. Petersson K. M. Hagoort P. (2013). The suppression of repetition enhancement: a review of fMRI studies. Neuropsychologia 51, 59–66. 10.1016/j.neuropsychologia.2012.11.006 23159344

[B73] Simons J. S. Koutstaal W. Prince S. Wagner A. D. Schacter D. L. (2003). Neural mechanisms of visual object priming: evidence for perceptual and semantic distinctions in fusiform cortex. Neuroimage 19, 613–626. 10.1016/S1053-8119(03)00096-X 12880792

[B74] Soldan A. Habeck C. Gazes Y. Stern Y. (2010). Neural mechanisms of repetition priming of familiar and globally unfamiliar visual objects. Brain Res. 1343, 122–134. 10.1016/j.brainres.2010.04.071 20450898 PMC2922055

[B75] Soldan A. Zarahn E. Hilton H. J. Stern Y. (2008). Global familiarity of visual stimuli affects repetition-related neural plasticity but not repetition priming. Neuroimage 39, 515–526. 10.1016/j.neuroimage.2007.08.011 17913513 PMC2140238

[B76] Tulving E. Schacter D. L. (1990). Priming and human memory systems.pdf. Science 247, 301–306. 10.1126/science.2296719 2296719

[B77] Turk-Browne N. B. Yi D. J. Chun M. M. (2006). Linking implicit and explicit memory: common encoding factors and shared representations. Neuron 49, 917–927. 10.1016/j.neuron.2006.01.030 16543138

[B78] Turkeltaub P. E. Eickhoff S. B. Laird A. R. Fox M. Wiener M. Fox P. (2012). Minimizing within-experiment and within-group effects in activation likelihood estimation meta-analyses. Hum. Brain Mapp. 33, 1–13. 10.1002/hbm.21186 21305667 PMC4791073

[B79] Wagner Anthony D. Koutstaal W. Maril A. Schacter D. L. Buckner R. L. (2000). Task-specific repetition priming in left inferior prefrontal cortex. Cereb. Cortex 10, 1176–1184. 10.1093/cercor/10.12.1176 11073867

[B80] Wagner A. D. Desmond J. E. Demb J. B. Glover G. H. Gabrieli J. D. E. (1997). Semantic repetition priming for verbal and pictorial knowledge: a functional MRI study of left inferior prefrontal cortex. J. Cogn. Neurosci. 9, 714–726. 10.1162/jocn.1997.9.6.714 23964594

[B81] Wig G. S. (2012). Repetition suppression and repetition priming are processing outcomes. Cogn. Neurosci. 3247–248. 10.1080/17588928.2012.689964 24171753

[B82] Wig G. S. Grafton S. T. Demos K. E. Kelley W. M. (2005). Reductions in neural activity underlie behavioral components of repetition priming. Nat. Neurosci. 8, 1228–1233. 10.1038/nn1515 16056222

[B83] Wiggs C. L. Martin A. (1998). Properties and mechanisms of perceptual priming. Curr. Opin. Neurobiol. 8, 227–233. 10.1016/S0959-4388(98)80144-X 9635206

[B84] Wright P. Randall B. Clarke A. Tyler L. K. (2015). The perirhinal cortex and conceptual processing: Effects of feature-based statistics following damage to the anterior temporal lobes. Neuropsychologia 76, 192–207. 10.1016/j.neuropsychologia.2015.01.041 25637774 PMC4582809

[B85] Yaple Z. Arsalidou M. (2017). Negative priming: A meta-analysis of fMRI studies. Exp. Brain Res. 235, 3367–3374. 10.1007/s00221-017-5065-6 28821983

[B86] Yi D.-J. (2005). Attentional modulation of learning-related repetition attenuation effects in human parahippocampal cortex. J. Neurosci. 25, 3593–3600. 10.1523/JNEUROSCI.4677-04.2005 15814790 PMC6725381

[B87] Zago L. Fenske M. J. Aminoff E. Bar M. (2005). The rise and fall of priming: how visual exposure shapes cortical representations of objects. Cereb. Cortex 15, 1655–1665. 10.1093/cercor/bhi060 15716471 PMC1564465

