# Supplementary material for: Neural Correlates of Repetition Priming: A Coordinate-Based Meta-Analysis of fMRI Studies
Source: Front Hum Neurosci. 2020 Sep 18;14:565114. doi: 10.3389/fnhum.2020.565114 (PMC7530292; doi:10.3389/fnhum.2020.565114)
Supplement: Supplementary file 1 [file Table_1.docx]

Supplementary Material

## Table S1. Summary of studies included in the meta-analysis.

| 1st author | Year | Subject | Stimulus | Task | Contrast | Threshold | Lag^a^ | Foci | Repetition effects | Process |
| --- | --- | --- | --- | --- | --- | --- | --- | --- | --- | --- |
| Badgaiyan | 2000 | 10 | Objects | Object identification | Priming > baseline | Uncorrected *p* < .001, cluster > 5 | Long | 1 | RS | Perceptual |
| Ballesteros | 2013 | 19 Young | Objects | Living/nonliving J. | Rep1 > rep2 | Uncorrected *p* < .001, cluster > 10 | Long | 16 | RS | Conceptual |
|  |  | 18 Old | Objects | Living/nonliving J. | Rep1 > rep2 | Uncorrected *p* < .001, cluster > 10 | Long | 10 | RS | Conceptual |
| Basso | 2013 | 10 | Objects | Naming^b^ | fMRI 1 > fMRI 2 | FDR-corrected *p* < .05, cluster > 80 | Long | 11 | RS | Perceptual |
|  |  | 10 | Objects | Naming | fMRI 1 < fMRI 2 | FDR-corrected *p* < .05, cluster > 80 | Long | 2 | RE | Perceptual |
| Bentley | 2003 | 30 | Face-face pairs | Same/different J. | First presentation > second presentation | Uncorrected *p* < .001, cluster > 5 | Long | 12 | RS | Perceptual |
|  |  | 30 | Face-face pairs | Same/different J. | First presentation < second presentation | Uncorrected *p* < .001, cluster > 5 | Long | 7 | RE | Perceptual |
| Bergerbest | 2009 | 16 Young | Words | Abstract/concrete J. | New > repeated | Uncorrected *p* < .001, cluster > 5 | Long | 3 | RS | Conceptual |
|  |  | 16 Old | Words | Abstract/concrete J. | New > repeated | Uncorrected *p* < .001, cluster > 5 | Long | 7 | RS | Conceptual |
| Binder | 2005 | 32 | Scenes | Indoor/outdoor J. | Novel > repeating | Uncorrected *p* < .05 | Long | 15 | RS | Conceptual |
| Blondin | 2005 | 24 | Scenes | Nature/human scene J. | First segment > second segment | Uncorrected *p* < .001, cluster > 5 | Long | 24 | RS | Conceptual^c^ |
| Buckner | 1998 | 20 | Objects | "Move on its own or not" J. | Novel > repeated | Uncorrected *p* < .001, cluster > 5 | Long | 14 | RS | Conceptual |
| Bunzeck | 2006 | 16 | Faces & Scenes | Male/female J. & Indoor/outdoor J. | New faces and scenes > old faces and scenes | Uncorrected *p* < .001 | Long | 9 | RS | Conceptual |
|  |  | 16 | Faces & Scenes | Male/female J. & Indoor/outdoor J. | New faces and scenes < old faces and scenes | Uncorrected *p* < .001 | Long | 4 | RE | Conceptual |
| Chao | 2002 | 7 | Objects | Naming^b^ | New > old | Uncorrected *p* < .05 | Long | 17 | RS | Perceptual |
|  |  | 7 | Objects | Naming | New < old | Uncorrected *p* < .05 | Long | 9 | RE | Perceptual |
| Chouinard | 2012 | 14 | Animals | 3 category classification | Non-repeated > repeated | Bonferroni-corrected p < .05 | Short | 20 | RS | Conceptual |
|  |  | 14 | Artifacts | 3 category classification | Non-repeated > repeated | Bonferroni-corrected p < .05 | Short | 11 | RS | Conceptual |
| Daselaar | 2005 | 25 Young | Words | Word stem completion | Primed > control | FDR-corrected *p* < .05, cluster = 5 | Long | 5 | RS | Perceptual |
|  |  | 25 Old | Words | Word stem completion | Primed > control | FDR-corrected *p* < .05, cluster > 5 | Long | 3 | RS | Perceptual |
| De Grauwe | 2014 | 21 | Words | Lexical decision | L2 unprimed > primed | Uncorrected *p* < .005, cluster > 65 | Short | 3 | RS | Perceptual |
|  |  | 21 | Words | Lexical decision | L2 unprimed < primed | Uncorrected *p* < .005, cluster > 65 | Short | 6 | RE | Perceptual |
| Donaldson | 2001 | 22 | Words | Abstract/concrete J. | New > old | Uncorrected *p* < .001, cluster > 5 | Long | 12 | RS | Conceptual |
|  |  | 22 | Words | Abstract/concrete J. | New < old | Uncorrected *p* < .001, cluster > 5 | Long | 30 | RE | Conceptual |
| Eger | 2004 | 13 | Mirrored objects | Size J. | baseline > primed attended | Uncorrected *p* < .001 (uncorrected *p* < .001 for mask contrast) | Short | 8 | RS | Conceptual^c^ |
| Eger | 2005 | 15 | Faces | Gender J. | Unprimed > same | Uncorrected *p* < .001 (uncorrected *p* < .001 for mask contrast) | Short | 2 | RS | Conceptual^c^ |
| Fiebach | 2005 | 14 | Words and pseudowords | Lexical decision | First presentation > second presentation | Cluster-level *p* < .05 (voxel-level uncorrected *p* < .0005) | Long | 7 | RS | Perceptual |
|  |  | 14 | Words and pseudowords | Lexical decision | First presentation < second presentation | Cluster-level p < .05 (voxel-level uncorrected p < .0005) | Long | 2 | RE | Perceptual |
| Friese | 2012 | 20 | Familiar objects | Familiar/unfamiliar J. | Initial > repeated | Cluster-level FWE-corrected *p* < .05 | Long | 7 | RS | Conceptual |
| Golby | 2005 | 7 | Scenes | Indoor/outdoor J. | Novel > repeat | Uncorrected *p* < .001, cluster > 5 | Short | 23 | RS | Conceptual |
| Gomes | 2016 | 18 | Object-object pairs | Size J. | New > recombined | Cluster-level *p* < .05 (voxel-level uncorrected *p* < .001, cluster > 21 determined by Monte Carlo simulation) | Long | 12 | RS | Conceptual |
|  |  | 18 | Object-object pairs | Size J. | New < recombined | Cluster-level *p* < .05 (voxel-level uncorrected *p* < .001, cluster > 21 determined by Monte Carlo simulation) | Long | 4 | RE | Conceptual |
| Graves | 2008 | 12 | Pseudowords | Naming^b^ | Decrease with number of repetitions | Cluster-level *p* < .05 (voxel-level uncorrected *p* < .001, cluster > 11 determined by Monte Carlo simulation) | Long | 5 | RS | Perceptual |
| Guitart-Masip | 2010 | 16 | Scenes | Indoor/outdoor J. | Novel > familiar | uncorrected *p* < .001 | Long | 72 | RS | Conceptual |
| Habeck | 2006 | 14 | Objects | Possible/impossible | Negative | Uncorrected *p* < .001 | Long | 14 | RS | Perceptual |
|  |  | 14 | Objects | Possible/impossible | Positive | Uncorrected *p* < .001 | Long | 12 | RE | Perceptual |
| Haist | 2001 | 15 | Words | Pseudohomophone-based Lexical decision | First presentation > second presentation | Bonferroni-corrected *p* < .05 | Long | 1 | RS | Perceptual |
|  |  | 15 | Words | Pseudoword-based Lexical decision | First presentation < second presentation | Bonferroni-corrected *p* < .05 | Long | 1 | RE | Perceptual |
| Hara | 2007 | 15 | Words | Lexical decision | First presentation > second presentation | Uncorrected *p* < .001 | Long | 2 | RS | Perceptual |
| Hawco | 2014 | 22 | Object triads | Size & related/unrelated J. | Novel > repeat | Cluster-level *p* < .05 (voxel-level uncorrected *p* < .001, cluster > 48 determined by Monte Carlo simulation) | Long | 14 | RS | Conceptual |
| Heath | 2012 | 18 | Objects | Naming^b^ | Unfacilitated > short-term | Cluster-level *p* < .05 (voxel-level uncorrected *p* < .001, cluster > 23 determined by Monte Carlo simulation) | Long | 1 | RS | Perceptual |
|  |  | 18 | Objects | Naming | Unfacilitated < short-term | Cluster-level *p* < .05 (voxel-level uncorrected *p* < .001, cluster > 23 determined by Monte Carlo simulation) | Long | 1 | RE | Perceptual |
| Henson | 2002 | 12 | Faces | Famous/nonfamous J. | First presentation > second presentation | Uncorrected *p* < .001 | Long | 2 | RS | Conceptual |
|  |  | 12 | Faces | Famous/nonfamous J. | First presentation < second presentation | Uncorrected *p* < .01, cluster > 10 | Long | 4 | RE | Conceptual |
| Henson | 2003 | 18 | Faces | Gender J. | Initial presentation > repeated presentation | Uncorrected *p* < .001, cluster > 10 | Long | 5 | RS | Conceptual |
| Heusser | 2013 | 16 | Objects | Man-made/natural J. | First > repeated | Uncorrected *p* < .001, cluster > 10 | Long | 5 | RS | Conceptual |
|  |  | 16 | Words | Man-made/natural J. | First > repeated | Uncorrected *p* < .001, cluster > 10 | Long | 4 | RS | Conceptual |
| Horner | 2008 | 18 | Objects | Size J. | Novel > repeated | RFT-corrected *p* < .05 | Long | 7 | RS | Conceptual |
|  |  | 18 | Objects | Size J. | Novel < repeated | RFT-corrected *p* < .05 | Long | 1 | RE | Conceptual |
| Korsnes | 2008 | 16 | Real objects | Real/unreal J. | New > old | Uncorrected *p* < .001 | Long | 9 | RS | Conceptual |
|  |  | 16 | Real objects | Real/unreal J. | New < old | Uncorrected *p* < .001 | Long | 3 | RE | Conceptual |
| Korsnes | 2014 | 16 | Objects | Real/unreal J. | New > old | Uncorrected *p* < .001 | Long | 4 | RS | Conceptual |
|  |  | 16 | Objects | Real/unreal J. | New < old | Uncorrected *p* < .001 | Long | 9 | RE | Conceptual |
| Kremers | 2014 | 23 | Face-building pairs | Fit/unfit J. | First presentation > second presentation | FWE-corrected *p* < .05, cluster > 20 | Long | 8 | RS | Conceptual |
|  |  | 23 | Face-building pairs | Fit/unfit J. | First presentation < second presentation | FWE-corrected *p* < .05, cluster > 20 | Long | 6 | RE | Conceptual |
| Kubicki | 2003 | 9 | Words | Abstract/concrete J. | New > same | Cluster-level *p* < .05 | Long | 1 | RS | Conceptual |
| Lin | 2007 | 17 | Mirror-reversed words | Mirror reading | Unidentified-prime control > unidentified-prime target | Uncorrected *p* < .001, cluster > 5 | Long | 21 | RS | Perceptual |
| Luo | 2004 | 9 | Words | Italic/upright J. | Unrelated > repetition | Uncorrected *p* < .01, cluster > 5 | Short | 1 | RS | Perceptual |
| MacDonald | 2015 | 18 | Objects | Naming^b^ | Unprimed > long-term | Uncorrected *p* < .001 and voxel-level FDR-corrected *p* < 0.05 | Long | 5 | RS | Perceptual |
|  |  | 18 | Objects | Naming | Unprimed < long-term | Uncorrected *p* < .001 and voxel-level FDR-corrected *p* < 0.05 | Long | 1 | RE | Perceptual |
| Manelis | 2011 | 14 | Objects | Natural/man-made | Presentation1 > 2 | Uncorrected *p* < .001 | Long | 13 | RS | Conceptual |
|  |  | 14 | Objects | Natural/man-made | Presentation1 < 2 | Uncorrected *p* < .001 | Long | 7 | RE | Conceptual |
| Poldrack | 1998 | 6 | Mirror-reversed words | Lexical decision | Unpracticed > practiced | Uncorrected *p* < .05 | Long | 16 | RS | Perceptual |
|  |  | 6 | Mirror-reversed words | Lexical decision | Unpracticed < practiced | Uncorrected *p* < .05 | Long | 7 | RE | Perceptual |
| Poldrack | 2001 | 16 | Mirror-reversed words | Lexical decision | Novel > repeated | Uncorrected *p* < .001, cluster > 10 | Long | 5 | RS | Perceptual |
| Poppenk | 2016 | 18 | Proverbs | Quality or target age rating | Novel > repetition & prior knowledge | *p* < .001 (bootstrap standard error) | Long | 8 | RS | Conceptual |
|  |  | 18 | Proverbs | Quality or target age rating | Novel < repetition & prior knowledge | *p* < .001 (bootstrap standard error) | Long | 5 | RE | Conceptual |
| Pourtois | 2005 | 14 | Faces | Gender J. | First > repeated (different views) | Uncorrected *p* < .05 (uncorrected *p* < .01 for mask contrast) | Long | 5 | RS | Conceptual^c^ |
| Qiao | 2014 | 17 | Words | Silent reading | Novel > repeated | Uncorrected *p* < .0005, cluster > 20 | Long | 2 | RS | Perceptual |
| Raposo | 2006 | 15 | Words | Abstract/concrete J. | Unrelated > identity | Cluster-level *p* < .05 (voxel-level uncorrected *p* < .01) | Short | 12 | RS | Conceptual |
|  |  | 15 | Words | Abstract/concrete J. | Unrelated < identity | Cluster-level *p* < .05 (voxel-level uncorrected *p* < .01) | Short | 6 | RE | Conceptual |
| Reber | 2005 | 9 | Objects | Target/nontarget J. | First presentation > second presentation | Uncorrected *p* < .01, cluster > 350 mm^3^ | Long | 14 | RS | Perceptual |
| Saggar | 2010 | 22 | Novel objects | Fat/slim J. | No-prime > low-prime | Cluster-level *p* < .05 | Long | 17 | RS | Perceptual |
| Schott | 2005 | 25 (Exp 1) | Words | Word stem completion | Correct rejections > primed items | Uncorrected *p* < .001, cluster > 5 | Long | 29 | RS | Perceptual |
|  |  | 16 (Exp 2) | Words | Word stem completion | Correct rejections > primed items | Uncorrected *p* < .001, cluster > 5 | Long | 32 | RS | Perceptual |
| Schwartz | 2013 | 8 | Faces | Gender J. | Initial > repeated | Cluster-level *p* < .05 (voxel-level uncorrected *p* < .005, cluster > 35 determined by Monte Carlo simulation) | Long | 3 | RS | Conceptual |
| Seger | 2000 | 7 | Nouns | Verb generation | Novel > repeated | Cluster-level *p* < .05 | Long | 10 | RS | Conceptual |
|  |  | 7 | Nouns | Verb generation | Novel < repeated | Cluster-level *p* < .05 | Long | 10 | RE | Conceptual |
| Simons | 2003 | 16 | Objects | Size J. | Novel > repeated | Cluster-level *p* < .05 (voxel-level uncorrected p < .001) | Long | 10 | RS | Conceptual |
| Soldan | 2008 | 14 | Familiar & unfamiliar objects | Real/unreal J. | First presentation > forth presentation | RFT-corrected *p* < .05, cluster > 5 | Long | 5 | RS | Conceptual |
|  |  | 14 | Familiar & unfamiliar objects | Real/unreal J. | First presentation < forth presentation | RFT-corrected *p* < .05, cluster > 5 | Long | 1 | RE | Conceptual |
| Soldan | 2010 | 14 | Familiar objects | Real/unreal J. | First > second presentation | Uncorrected *p* < .001, cluster > 50 | Long | 23 | RS | Conceptual |
|  |  | 14 | Unfamiliar objects | Real/unreal J. | First < second presentation | Uncorrected *p* < .001, cluster > 50 | Long | 6 | RE | Conceptual |
| Thiel | 2001 | 13 | Words | Word stem completion | Placebo new > old | Uncorrected *p* < .001 | Long | 10 | RS | Perceptual |
| Turk-Browne | 2006 | 16 | Scenes | Indoor/outdoor J. | First > second exposure | Uncorrected *p* < .001, cluster > 5 | Long | 5 | RS | Conceptual |
| Turk-Browne | 2010 | 16 | Faces & scenes | Face/scene J. | Unprimed > second | Cluster-level *p* < .05 (voxel-level uncorrected *p* < .001, cluster > 5) | Long | 1 | RS | Conceptual |
| van Turennout | 2003 | 10 | Objects | Naming^b^ | Novel > single repetition | Uncorrected *p* < .05 (uncorrected *P* < 0.005 for mask contrast) | Long | 6 | RS | Perceptual |
|  |  | 10 | Objects | Naming | Novel < single repetition | Uncorrected *p* < .05 (uncorrected *P* < 0.005 for mask contrast) | Long | 2 | RE | Perceptual |
| Voss | 2008 | 11 | Faces | Fame J. | Unprimed > primed | Cluster-level *p* < .01 (voxel-level uncorrected *p* < .01, cluster > 12 determined by Monte Carlo simulation) | Long | 2 | RS | Conceptual |
|  |  | 11 | Faces | Fame J. | Unprimed < primed | Cluster-level *p* < .01 (voxel-level uncorrected *p* < .01, cluster > 12 determined by Monte Carlo simulation) | Long | 4 | RE | Conceptual |
| Vuilleumier | 2002 | 12 | Real and non-objects | Real/unreal J. | New > repeated | Uncorrected *p* < .001 | Long | 2 | RS | Conceptual^c^ |
| Vuilleumier | 2005 | 10 | Objects | Real/unreal J. | New > old attended | Uncorrected *p* < .001 | Long | 9 | RS | Conceptual |
| Wagner | 2000 | 10 | Words | Abstract/concrete J. | Novel > repeated: within-task | Uncorrected *p* < .001, cluster > 5 | Long | 9 | RS | Conceptual |
| Wang | 2014 | 21 | Words | Free association | Unprimed + baseline > primed | Cluster-level *p* < .05 (voxel-level uncorrected *p* < .001, cluster > 20) | Long | 32 | RS | Conceptual |
| Wheatley | 2005 | 15 | Words | Silent reading | Unrelated > identical | Uncorrected *p* < .001 (uncorrected *P* < 10-6 for mask contrast) | Short | 13 | RS | Perceptual |
| Yang | 2008 | 13 | Word-word pairs | Silent reading | New > old & recombined | Uncorrected *p* < .05 (uncorrected *P* < 10-4 for mask contrast) | Long | 10 | RS | Perceptual |
| Zago | 2005 | 12 (Exp 1) | Objects | Natural/manufactured J. | New > repeat | Uncorrected *p* < .001 | Long | 7 | RS | Conceptual |
|  |  | 12 (Exp 2) | Objects | Natural/manufactured J. | New > repeat | Uncorrected *p* < .001 | Long | 7 | RS | Conceptual |
|  |  | 12 (Exp 1) | Objects | Natural/manufactured J. | New < repeat | Uncorrected *p* < .001 | Long | 4 | RE | Conceptual |
|  |  | 12 (Exp 2) | Objects | Natural/manufactured J. | New < repeat | Uncorrected *p* < .001 | Long | 4 | RE | Conceptual |

^a^The length of the lag between initial and primed stimuli is classified as “long” if there are more than one intervening stimuli and as “short” if there no intervening stimuli.

^b^Six naming experiments were excluded in a sub-analysis.

^c^Five experiments without perceptual overlap were included in a sub-analysis.

RS, Repetition Suppression; RE, Repetition Enhancement; J., Judgments; Exp, Experiment
